# Development in Rice Genome Research Based on Accurate Genome Sequence

**DOI:** 10.1155/2008/348621

**Published:** 2008-06-18

**Authors:** Takashi Matsumoto, Jianzhong Wu, Baltazar A. Antonio, Takuji Sasaki

**Affiliations:** Division of Genome and Biodiversity Research, National Institute of Agrobiological Sciences, 2-1-2, Kannondai, Tsukuba, Ibaraki 3058602, Japan

## Abstract

Rice is one of the most important crops in the world. Although genetic improvement is a key technology for the acceleration of rice breeding, a lack of genome information had restricted efforts in molecular-based breeding until the completion of the high-quality rice genome sequence, which opened new opportunities for research in various areas of genomics. The syntenic relationship of the rice genome to other cereal genomes makes the rice genome invaluable for understanding how cereal genomes function. Producing an accurate genome sequence is not an easy task, and it is becoming more important as sequence deviations among, and even within, species highlight functional or evolutionary implications for comparative genomics.

## 1. INTRODUCTION

Food security is a major issue as we aspire toward sustainable development. In spite of continuous increases in agricultural production due to the introduction of improved crop cultivars and the wide use of affordable technologies, more than 800 million people still do not have access to sufficient food to meet their dietary needs [1]. Cereal crops are basic source of food for humankind, with 85% of total crop production represented by maize, wheat, and rice. These three crops provide more than half of the protein and energy required for daily life. However, increase of world agricultural production in 2006 was less than 1%, which was due to decrease in cereal production [2]. On the other hand, the world’s population is expected to reach 9 billion by 2050 [3]. It is therefore necessary to provide food security to this growing population in the midst of global environmental problems that deprive us of much arable land and biodiversity.

Worldwide transformation of agriculture was first achieved with the Green Revolution, which led to significant increases in agricultural production. It began in the 1940s with the cultivation of a high-yielding dwarf wheat cultivar with resistance to pests and diseases. The Green Revolution for rice in the 1960s, based on the cultivar IR8, also dramatically increased rice production and helped food production to keep pace with population growth.

Now, the second Green Revolution, which will be based on genomics, is expected to pave the way for the leap in crop production. The availability of the rice genome sequence allowed the development of innovative approaches to increasing production. In the last 10 years, the basic syntenic relationships in gene content and gene order within the grass family have been established [4–6]. Therefore, the rice genome could be used as a reference genome for understanding the evolution of cereal crops and could provide a basis for their improvement [7, 8].

Among plants, only the Arabidopsis [9] and rice [10] genome sequences have been completed so far. A positionally confirmed, quality-validated genome sequence is obligatory required as a reference for the efficient use of sequence information, particularly in comparative analysis. Hence, the genome sequence derived from *Oryza* *sativa* ssp. *japonica* cv. Nipponbare has been recognized as a gold standard for understanding the genetics and biology of rice at the molecular level and in the breeding and genetic manipulation of cereal crops.

This chapter presents a past history of the rice genome sequencing efforts and a present endeavor for analysis of the genome sequence to clarify its structure and function. Approach to the “difficult” regions whose functions are the maintenance and regulation of chromosomes—notably the centromeres and telomeres—is described. Application of the new sequencing technology toward comparative studies among genus *Oryza* is also described in the context of the rice genome as a reference.

## 2. GENOME SEQUENCING THROUGH INTERNATIONAL COLLABORATION

**Figure 1:**
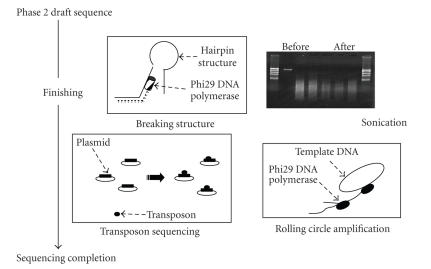
Four steps used for the finishing process to sequence completion.

The International Rice Genome Sequencing Project (IRGSP) was established in 1997. The 10 member countries agreed to sequence a standard rice cultivar (Nipponbare), to use common resources, and to share sequencing of the 12 rice chromosomes by using a map-based clone-by-clone strategy (http://rgp.dna.affrc.go.jp/E/IRGSP/index.html). For construction of sequence-ready physical maps, two complementary approaches were used. The Rice Genome Research Program (RGP) in Japan anchored the genomic clones using expressed sequence tags/sequence-tagged sites (EST/STS) and genetic markers from the genetic and transcript maps of rice [11, 12]. The Clemson University Genomics Institute, the Arizona Genomics Institute, and the Arizona Genomics Computational Laboratory used a high-throughput bacterial artificial chromosome (BAC) fingerprint and automatic BAC contig assembly system using FPC software [13], and anchored the assembled contigs on the rice genome by hybridization-based screening [14]. The sequence-ready physical maps generated from use of these two strategies covered more than 95% of the rice genome, and 92% to 100% of each chromosome. A total of 3453 PAC/BAC clones forming the minimum tiling path were selected for sequencing. DNA from a BAC/PAC clone was purified and fragmented by sonication. The ends of 2000 subclones of each clone were sequenced with capillary sequencers and assembled using the phred/phrap assembler [15]. The genome sequences of each PAC/BAC clone at the high-throughput genomic (HTG) phase 2 category were submitted to the DNA Data Bank of Japan (DDBJ). By December 2002, almost all the clone sequences corresponding to the minimum tiling path were sequenced to at least HTG phase 2. As a result, a high-quality draft sequence representing 366 Mb of the rice genome was released in the public database [16]. Thereafter, the IRGSP continued with the arduous task of finishing: gap-filling, improving base read quality, and resolving misassemblies (Figure 1).

In December 2004, the high-quality map-based sequence of the rice genome at HTG phase 3 category was completed and released in the public domain [10]. The sequence, ca. 370 Mb in total, covered nearly 95% of the total estimated size of the genome and about 99% of the euchromatic regions. The sequence also included three centromeres, parts of the rDNA regions, and regions for various transposable elements (corresponding up to 35% in the total genome). This comprehensive, relatively accurate sequence of the rice genome, is currently considered the gold standard.

In contrast to the hierarchical clone-by-clone strategy used by the IRGSP, a whole-genome shotgun (WGS) sequencing strategy is widely used in many sequencing projects [17]. In this strategy, a high-throughput computer program to reproduce the entire genome sequence assembles millions of shotgun sequences from the total genome. This method was used in sequencing the 2.9-Gb human genome [18]. Two independent groups used the WGS strategy to sequence the rice genome. The Beijing Genome Institute assembled shotgun sequences of the *indica* line 93-11 with 4× [19] and later 6× [20] genome coverage. A private company, Syngenta (Basel, Switzerland), also used the WGS strategy to sequence the Nipponbare [21]. This WGS sequence of Nipponbare was further improved by reassembling the shotgun sequences and combining the *japonica* and *indica* (line 99-11) sequences, resulting in 433 Mb of sequence composed of 50 233 contigs of Nipponbare [20]. Nearly 99% of the rice full-length cDNAs [22] have been localized in these latest assemblies [20] of the *japonica* and *indica* genome.

The effectiveness of the WGS sequencing strategy was compared with that of the hierarchal clone-by-clone sequencing approach [23, 24]. Although WGS assembly could readily provide an overview of the genome structure with a practical level of accuracy, misassembly could result in nonhomologous, misaligned, or duplicated coverage and some mismatches even in the genic regions. Moreover, repeat sequences could not be properly assigned to their original positions in the genome. In the case of rice, which has a lot of repeat sequences, WGS sequencing is therefore not a highly reliable strategy as it creates misassembly, particularly in duplicated regions. It is therefore important to have a highly accurate map-based sequence, which can be obtained by the hierarchical clone-by-clone strategy. Today projects aiming at obtaining entire genome sequences of gramineae plants are progressing [25–29]. All the projects, either using WGS strategy or clone-by-clone strategy, regard the completed rice genome sequence as sequence reference in reconstruction of chromosome sequences, emphasizing the importance of “gold standard.”

## 3. DECIPHERING THE GENOME THROUGH ANNOTATION

**Figure 2:**
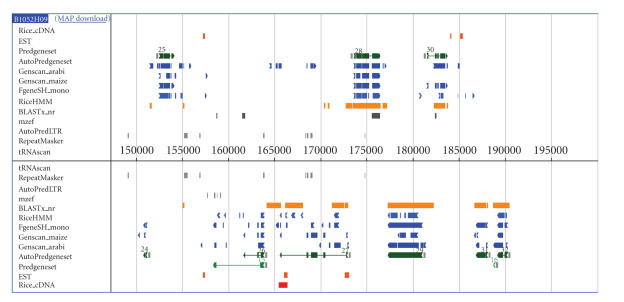
RiceGAAS annotation view, showing results from application of gene prediction software and similarity searches. Upper box: a DNA strand from left (5′) to right (3′). Lower box: from right (5′) to left (3′).

Detecting the gene-coding regions within the genome sequence is one of the most efficient ways to characterize the structure and function of the genome. RGP constructed an annotation system that facilitates gene detection of the genome sequence in a timely manner. The Rice Genome Automated Annotation System, or RiceGAAS (http://ricegaas.dna.affrc.go.jp [30]), was designed as a fully automated system for annotating rice genome sequences. It retrieves rice sequences from GenBank and analyzes them with gene prediction programs such as Genscan [31] and FgeneSH (http://www.softberry.com/berry.phtml) and with BLAST [32] for similarity to proteins, rice ESTs, and rice full-length cDNAs to generate the most accurate gene models on the basis of available information (Figure 2). A similar automatic annotation pipeline was established by TIGR (http://rice.tigr.org/tdb/e2k1/osa1/data_download.shtml), and gene models are improved with rice ESTs and transcripts [33]. Both sets of gene models are published on the Web to accelerate gene analysis. With increasing data on nucleic acids and proteins in the public databases, regular re-evaluation and update of these gene models is necessary. In this respect, one of the advantages of these full-computational approaches is that whole gene sets can be relatively easily revised.

RGP has also developed a manual annotation system to facilitate curation of the gene models by human annotators (http://rgp.dna.affrc.go.jp/genomicdata/AnnSystem.html). This pipeline directly takes the output generated from RiceGAAS for in-depth analysis with in-house editing tools. Each gene model is manually edited to improve the prediction accuracy. The gene models for each BAC or PAC clone are released to the public domain through the DDBJ/EMBL/GenBank database. All data can be accessed through the central database whole genome annotation (WhoGA) on our website at http://rgp.dna.affrc.go.jp. Initially, only the six chromosomes (1, 2, 6, 7, 8, and 9) assigned to RGP were manually curated. Recently, curation of the rest was completed, so the manual annotation of the entire genome is now available. After removal of clone overlaps, a total of 57 724 genes were predicted, including many hypothetical genes predicted by a single prediction program. Among them, 24056 gene models are supported by full-length cDNAs. All the gene models are ordered and organized in a genome browser.

Apart from these individual activities, the IRGSP conceived the establishment of the Rice Annotation Project (RAP), a community standard annotation project, in 2004. Genes were annotated at regular jamboree-style annotation meetings to facilitate the manual curation of all gene models in rice. The National Institute of Agrobiological Sciences has been leading this project, collaborating with IRGSP members and many international and Japanese laboratories. So far, three RAP meetings have been held, at which gene models, chiefly constructed by mapping full-length cDNAs on the latest rice genome assemblies, have been manually curated. This collaboration confirmed 32000 curated genes, most of which have some degree of evidence [34]. The RAP-database (RAP-DB, http://rapdb.lab.nig.ac.jp) will be further improved with the integration of other annotation and functional genomics data.

## 4. UNCOVERED TERRITORY—EXPLORATION OF THE MISSING REGIONS

At the time of completion of the genome in 2004, IRGSP published nearly 371 Mb of high-quality DNA sequences, leaving about 5% of its estimated 389 Mb to be sequenced [10]. These unsequenced genomic regions existed as 62 gaps, including the telomeres and centromeres in all but two out of 12 chromosomes. One of the main reasons for the presence of these gaps was that no more clones with sequence extension into gap regions could be selected from any Nipponbare genomic resources, including BAC and PAC libraries (both based on partial digestion of DNA fragments) and fosmid libraries (based on physically sheared DNA fragments), containing a total of 630 000 clones. For unknown reasons, specific genomic regions could not be cloned or maintained by using the above vectors in bacteria. In addition, a number of regions in the genome contain highly repeated sequences, making it difficult to construct a correct and complete physical map. However, analysis of sequences from these complicated genomic regions is not futile. Researchers have reported the importance of heterochromatic regions in silencing gene expression [35]. Cytological analysis has been used to define the distribution of such heterochromatin along each rice chromosome [36]. Through the IRGSP efforts, 2 of the 12 centromeres and 14 of the 24 telomeres have been completely or partly sequenced (http://rgp.dna.affrc.go.jp/E/IRGSP/index.html). Here, we focus on both regions because they play essential roles in chromosome maintenance or segregation.

### 4.1. Composition and structure of rice centromeres

**Figure 3:**
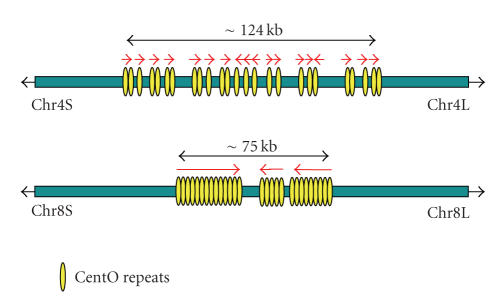
Structural comparisons of *CentO* domains between Nipponbare chromosomes 4 and 8. Yellow ovals and red arrows indicate the position of *CentO* arrays and the direction of the 155-bp tandem repeats within each array, respectively. Length of arrays ranges from 477 to 8571 bp in chromosome 4 and 7616 to 34589 bp in chromosome 8.

Because of the relatively small amount of centromeric satellite DNA in rice, significant progress has been made in genomic and molecular studies of the structures, functions, and evolution of rice centromeres. Two centromeres, derived from chromosomes 4 and 8, have been completely sequenced, revealing the complicated composition and structure of the first centromeres to have been sequenced among eukaryotes [37–39]. Repetitive sequences occupy ~60% of the whole region (~2 Mb) of the centromere of chromosome 8 (*Cen8*). The majority of copies of the 155-bp centromeric satellite repeat *CentO*, totaling 68.5-kb, occur in three large clusters in the center, separated by centromere-specific retrotransposon of rice (*CRR*) sequences. Numerous sequences of other transposable elements were also found in its surrounding region. *Cen8* contains an ~750-kb core domain that binds rice CENH3, the centromere-specific H3 histone [37]. It is surprising to find transcriptionally active genes even within the core domain of *Cen8*. A similar result was found in *Cen3*, where a much bigger region (~1881 kb) has been found to have associations with CENH3 [40]. As a chromosomal site for kinetochore assembly that plays an important role in the faithful segregation of sister chromatids during cell division, the centromere has functions that are well conserved among all higher eukaryotes. Inter- and extrachromosomal analysis of the centromeres has, however, revealed the divergence of DNA components and organization patterns even among closely related species. The amount of *CentO* satellite DNA in the centromere of individual chromosomes varies from 60 kb to 1.9 Mb in *O. sativa* [41]. The number and organization of *CentO* clusters within the core region differ markedly between *Cen4* and *Cen8* in the Nipponbare genome. *Cen8* has only three *CentO* tracts (clusters) with 442 copies of the 155-bp tandem repeat distributed within a 75-kb region, whereas *Cen4* has up to 18 tracts but only 379 copies of the repeat within a 124-kb region [38, 39] (Figure 3). *CentO* repeats, on the other hand, are absent from several wild rice species, such as *Oryza brachyantha* [42]. It would be interesting to sequence and compare the compositional and structural changes in centromeres between different *Oryza* species in the future, since in-depth analysis of the *Cen8* and *Cen4* sequences has demonstrated segmental duplication and inversion of centromeric DNA [43]. First glimpse of this analysis was performed in sequencing the centromere region of chromosome.8 from *O. brachyantha,* revealing positional shift of centromere [44].

Rice is now becoming a model for centromere and heterochromatin research [38, 45, 46]. Further research will lead to insights into the evolutionary dynamics, processes, and molecular mechanisms of plant centromeres.

### 4.2. Composition and structure of rice telomeres

Like those of centromeres, the composition and structure of telomere regions in rice have also been analyzed. Telomeres form the ends of linear eukaryotic chromosomes, serving as protective caps that prevent end-to-end fusion, recombination, and degradation of chromosomal ends [47]. The telomeres of most eukaryotes consist of an array of repeats that contain similar sequences but vary in length. For example, telomere DNA has a conserved sequence of 5′-TTAGGG-3′ in humans and 5′-TTGGGG-3′ in *Tetrahymena* (a ciliate protozo) [48, 49]. The first plant telomere DNA was isolated from *Arabidopsis thaliana* and shows tandemly repeated arrays of 5′-TTTAGGG-3′ [50]. Rice telomeres consist of the same repeat [51]. Sequencing and extensive analysis of seven rice chromosomal ends revealed several basic features that could provide a platform for analyzing and understanding the telomere structures and functions. All seven rice telomeres revealed contain highly conserved TTTAGGG sequences in tandem repeats, although deletions, insertions, and substitutions of single nucleotides or inverted copies were found within the arrayed repeats, particularly in the region of the junction between the telomere and subtelomere. Fluorescent in situ hybridization and terminal restriction fragment analyses suggest that the rice telomeres are a bit longer than those of *Arabidopsis* but much shorter than those of *Nicotiana tabacum*, ranging in a length from 5 to 20 kb, thus hinting at the genetic control of telomere length in plants [52, 53]. Interestingly, variation in telomere length is observed not only among different chromosomes, but also between different species within *Oryza*; this variation should provide useful information for future studies of telomere evolution. Gene annotation in the 7 rice subtelomere regions (each within 500 kb) demonstrated that the genomic region adjacent to the chromosome terminus is gene-rich (1 gene per 5.9 kb on average). Since nearly half of these annotated genes match rice full-length cDNAs, these rice subtelomeres could be considered to have high transcriptional activity. Recently, seven new rice telomeres were partly sequenced, and their sequences have been submitted to DDBJ (Table 1; http://rgp.dna.affrc.go.jp/E/publicdata/telomere2007/index.html). Among the above 14 chromosomal ends, the telomere and subtelomere regions on the short arm of chromosome 9 show some specific compositional and structural features. Sequencing and analysis of the fosmid clone OSJNOa063K24 revealed that the telomere repeats are colocalized with the ribosomal RNA gene (rDNA) cluster [54]. Besides the telomere-specific repeat and the long rDNA array (sized in megabases), the content of repetitive sequences such as retrotransposons within the 500-kb region proximal to the centromere is relatively high, suggesting that much of the short arm of rice chromosome 9 is heterochromatic. Rice telomere reverse transcriptase has also been isolated [55]. It will be interesting to conduct future studies using rice as a model of telomere research, as has been done for centromeres, especially to reveal how telomere length (shortening or elongation) is regulated and whether the telomere repeats and structure affect the expression of genes in the subtelomere region. The sequence resources obtained from the telomere and centromere regions of rice chromosomes should thus provide an unprecedented opportunity for future study, particularly to construct an artificial chromosome for use in both molecular and applied biology in plant science.

## 5. GENOME SEQUENCE FOR EVOLUTIONARY GENOMICS IN RICE

**Figure 4:**
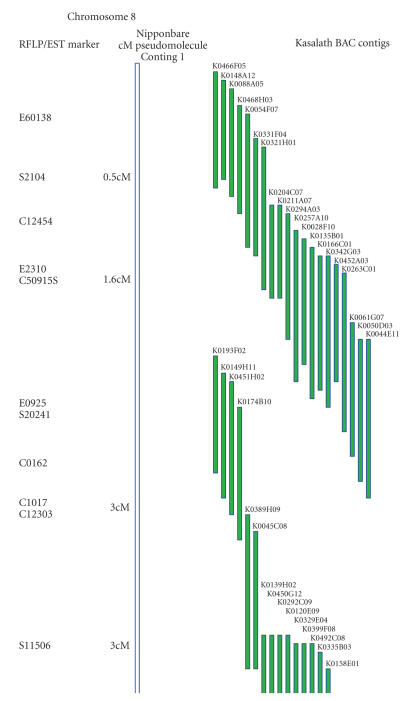
In silico physical map of Kasalath chromosome 8, based on the Nipponbare sequence. Green vertical bars indicate BAC clones (with K numbers) mapped against Nipponbare genome sequence (shown at left with landmarks).

Rice is believed to have been domesticated from a wild relative 0.2 Mya [56] or 0.44 Mya [57]. Asian cultivated rice (*O. sativa* L.) has two subspecies, *indica* and *japonica*. Both are important as modern crops, and there are many phenotypic variations among them, conferring adaptation to many different environmental and cultural conditions. Crossing of these subspecies has produced new cultivars of agricultural importance. Knowing the differences at the molecular level would widen the capacity for rice breeding. RGP constructed a BAC library of Kasalath, an *indica* cultivar, generating 78427 high-quality BAC end sequences from 47194 BAC clones, and mapped these end sequences on Nipponbare chromosome sequences [58]. Mapping of 12170 clones allowed the construction of 450 Kasalath BAC contigs covering 308.5 Mb. Single-nucleotide polymorphism (SNP) frequency in the BAC end sequences and corresponding Nipponbare sequences was 0.71% on average. Sequencing of part of the Kasalath genome is in progress and could in future elucidate the precise gene dynamics in evolution and domestication. Results of Kasalath BAC physical maps are shown on RGP homepage (http://rgp.dna.affrc.go.jp/E/publicdata/kasalathendmap/index.html). Figure 4 is an example of a computer-generated Kasalath BAC physical map. BLAST searches for Kasalath BAC-end sequence screening could be performed through website (http://rgp.dna.affrc.go.jp/blast/runblast.html). Other approaches [59, 60] could identify positions of SNPs for high-density SNP markers.

It had long been a mystery how Asian rice originated from its wild progenitor, *Oryza rufipogon*. Recently, the origin has been clarified by comparison of retrotransposon [56], retroposon [61], chloroplast [62], and gene [63] sequences along the evolutionary lineages. These studies show evidence of multiple independent domestications of the two major subspecies. Further molecular studies of domestication will show how the crop and humans coevolved.

The genus *Oryza* has 23 species [64], but only two species (*O. sativa* in Asia and *O. glaberrima* in Africa) are domesticated and cultivated. This fact is remarkable given that rice grows under a wide variety of natural conditions. Consequently, many genetic resources might be waiting to be developed. Study of the wild relatives might reveal new genes for hybridization, improved yield, and sustainable production. The *Oryza* Map Alignment Project (OMAP, http://www.omap.org/index.html) of the USA and China aims at the establishment of an experimental platform to unravel and understand the evolution, physiology, and biochemistry of the genus. The Arizona Genomics Institute has constructed 12 BAC libraries from the AA (the same as sativa species) to HHKK (remote species from sativa) species genomes. Computer-based mapping and filter hybridization screening provided high-density cross-species physical maps [65, 66].

## 6. IMPACT OF NEW SEQUENCING TECHNOLOGIES

The genome sequences of *O. sativa* and its progenitors are expected to show extensive base substitutions and rearrangements. Therefore, it would be difficult to reconstruct the genome sequences of wild rice relatives from cultivars. As resequencing with the conventional Sanger methodology can take much time and effort, a new pyrosequencing technology was developed. Massively parallel short reads from pyrosequencing analysis [67] could sequence more than 20 million bases with much less cost and less time than with Sanger analysis. In collaboration with 454 Life Sciences and Roche Diagnostics, we compared pyrosequencer and Sanger sequence data. Eight BAC clones which include OR_CBa0076I05, OR_CBa0091G05, OR_CBa0094N06, OR_CBa0004O24, OR_CBa0063M01, OR_CBa0075G04, OR_CBa0034E23, and OR_CBa0010H05 from *O. rufipogon* IRGC105491 (AA species) were chosen from a fingerprint contig of the OMAP BAC library (OR_CBa-FPC contig 51). This contig corresponds to an 800-kb region of the short arm of Nipponbare chromosome 6 and is expected to contain two genes for rice flowering (*Hd3a* and *RFT1*). DNA of each BAC clone was purified individually and then mixed for pyrosequencing on a GS20 genome analyzer (Roche). The output from this analysis (ca. 20× coverage) contained 286639 reads. Of these, 169130 reads were mapped and 16123462 bases were aligned to the corresponding Nipponbare sequences, forming 1422 mapped contigs that cover 57.5% of the entire genomic region. The average depth was 23.39 showing deep coverage.

To compare these sequences with those from Sanger sequencing, we shotgun sequenced a BAC clone OR_CBa0004O24 and assembled it with phred/phrap software to form contigs. Each contig sequence from pyrosequencing was aligned to its corresponding Sanger sequence by BLAST alignment. Statistical results from this comparison are shown in Table 2.

Comparing only high quality (HQ sequence quality score > either 30 or 40) nucleotides gave an overall error rate of 0.0409% or 0.0359%. This means that the high-coverage reads from pyrosequencing show more than 99.95% accuracy. Researchers have pointed out that pyrosequencing is more problematic in repeats and homopolymers than Sanger technology [68, 69], but we did not observe this type of discrepancy. We also compared nucleotide sequences of *Hd3a* (one of the rice heading date QTL, corresponding to FT gene of Arabidopsis) between *O. sativa* cv. Nipponbare (by Sanger method) and *O. rufipogon* (by pyrosequencing). Only 3 SNPs and no in/del were found in exons (540 coding nt), whereas many deviations (20 SNPs, 6 indels) were found in introns; this was evolutionally reasonable. This sequence conservation might indicate that *Hd3a* is functionally important and under purifying selection.

These results show that emerging new resequencing technologies (not only pyrosequencing but also other methods [70]), when properly used in combination with current methods, will revolutionize the cost and performance of rice genome resequencing and will help elucidate the evolution of the *Oryza* genomes.

## 7. CONCLUSION

The rice genome sequence has become available as a reference genome, providing a basis for understanding the wide range of diversity among cultivated and wild relatives of rice. The continuous efforts in generating a high-quality sequence have paved the way for clarifying the structures of genomic regions that are difficult to analyze, including centromeres and telomeres. Comparative genomics within the genus *Oryza* has also become a feasible strategy for understanding the evolutionary events that led to the development of cultivated rice. The syntenic relationships among cereal crops must be thoroughly exploited from now on. The rice genome sequence will be the most important tool in explaining the structure and function of other cereal genomes, and its use may open new opportunities for researchers to look deeper into the synteny between rice and other cereal crops, which has been maintained for some 60 million years of evolution [6]. From a more practical aspect, the rice genome sequence could be the key for developing rice-genomics-based research in order to improve crop production and food security for humankind.

## Figures and Tables

**Table 1:** Mapped and sequenced rice telomeres.

Clone name	Accession no.	Copies*	Chr
OSJNOa264G09	AP008219	17	1S
OSJNOa183H18	AP006851	52	2S
OSJNOa246I10	AP008220	69	2L
OSJNOa070P15	AP009053	27	3S
OSJNOa083A04	AP009055	75	3L
OSJNOa076I12	AP009056	129	4S
OSJNOa281H13	AP009057	68	4L
OSJNOa070B13	AP009052	53	5S
OSJNOa230J22	AP006854	37	6L
OSJNOa219C16	AP008222	17	7S
OSJNOa136M23	AP008223	127	7L
OSJNOa162K02	AP008224	55	8S
OSJNOa063K24	AP009051	162	9S
OSJNOa073B23	AP009054	62	10S

***Copies of telomere-specific repeats detected from the sequenced clones.

**Table 2:** Sequence comparison of BAC clone OR_CBa0004O24, Sanger versus Pyrosequencing.

	Low-quality threshold	
	Score 30	Score 40
Number of alignments checked	34	34
Total length of alignments	132229	132229
Total HQ bases	131759	130639
Total LQ bases	470	1590
“In/del” type discrepancy	20	20
* *– Sanger insertion, total	15	15
* *– Pyro insertion, total	5	5
LQ insertion, total	5	6
* *– Sanger LQ	3	3
* *– Pyro LQ	2	3
HQ insertion, total	15	14
* *– Sanger HQ insertion	12	12
* *– Pyro HQ insertion	3	2
“SNP” type discrepancy	60	60
* *– both HQ	54	47
* *– LQ for Sanger	5	12
* *– LQ for Pyro	1	1
* *– both LQ	0	0
Discrepancy rate (%)	0.0409	0.0359
Accuracy rate (%)	99.9591	99.9641
